# Accuracy of a Method to Monitor Root Position Using a 3D Digital Crown/Root Model during Orthodontic Treatments

**DOI:** 10.3390/tomography8020045

**Published:** 2022-02-23

**Authors:** Kaho Ogawa, Yoshiki Ishida, Yukinori Kuwajima, Cliff Lee, Jacob R. Emge, Mitsuru Izumisawa, Kazuro Satoh, Shigemi Ishikawa-Nagai, John D. Da Silva, Chia-Yu Chen

**Affiliations:** 1 Department of Oral Medicine, Immunity and Infection, Harvard School of Dental Medicine, 188 Longwood Avenue, Boston, MA 02115, USA; kaho_ogawa@hsdm.harvard.edu (K.O.); yishida@tky.ndu.ac.jp (Y.I.); ykuwaji@iwate-med.ac.jp (Y.K.); cliff_lee@hsdm.harvard.edu (C.L.); jacobemge@g.ucla.edu (J.R.E.); shigemi_nagai@hsdm.harvard.edu (S.I.-N.); 2 Division of Orthodontics, Department of Developmental Oral Health Science, School of Dentistry Iwate Medical University, 1-3-27 Chuo-dori, Morioka 020-8505, Iwate, Japan; kazsatoh@iwate-med.ac.jp; 3 Department of Dental Materials Science, School of Life Dentistry at Tokyo, Nippon Dental University, 1-9-20, Fujimi, Chiyoda-ku, Tokyo 102-8159, Japan; 4 Division of Dental Radiology, School of Dentistry, Iwate Medical University, 1-3-27 Chuo-dori, Morioka 020-8505, Iwate, Japan; mizumisa@iwate-med.ac.jp; 5 Department of Restorative Dentistry and Biomaterials Science, Harvard School of Dental Medicine, 188 Longwood Avenue, Boston, MA 02115, USA; john_dasilva@hsdm.harvard.edu

**Keywords:** digital dentistry, CBCT, digital scans, orthodontic tooth movement

## Abstract

This study aimed to assess the accuracy of a method of predicting post-movement root position during orthodontic treatment using a 3D digital crown/root model (3DCRM) created with pre-movement records of both cone-beam computed tomography (CBCT) and dental arch digital scans. Pre- and post-movement CBCT scans and dental arch digital scans of five patients who had completed orthodontic treatments were used in this study. The 3DCRM was superimposed onto the post-movement scanned dental arch to identify the post-movement root position (test method). Post-movement CBCT (referenced as the current method) served as the control to identify the actual post-movement root position. 3D-coordinate analysis revealed no significant differences between the test and current methods along the X and Y axes. However, the discrepancy on the Z axis (especially in cases of intrusion) was greater than that in all other directions for all three tooth types examined (*p* < 0.05). A strong positive correlation between the degree of discrepancy and the distance of tooth movement was observed on the Z axis (*r* = 0.71). The 3DCRM method showed promising potential to accurately predict root position during orthodontic treatments without the need for a second CBCT. However, root resorption, which affected the Z axis prediction, needs to be closely monitored using periapical radiographs to complement this method.

## 1. Introduction

The goal of orthodontic treatment is to establish ideal three-dimensional crown and root position in a functional, stable, and esthetic occlusion. Andrews reported six keys to normal occlusion, (1) molar relationship, (2) crown angulation (mesiodistal tip), (3) crown inclination (labiolingual or buccolingual inclination), (4) rotations, and (5) occlusal plane and (6) occlusion based on crown information from study models [1]. Proper root position and parallelism are imperative for adequate occlusal function, periodontal health, implant placement, and restorative treatment [2,3,4,5]. Root position and parallelism are important factors for achieving even distribution of occlusal forces to create an ideal function and for establishing proper contours and emergence profiles of restorations [2,3,4,5]. Root proximity may lead to rapid periodontal breakdown and horizontal bone loss instead of intrabony defects that are amenable to regeneration [6,7,8,9,10,11]. The consequences of roots moving out of the alveolar housing include clinical attachment loss, recession, bone dehiscence, mobility, and even tooth loss [12,13]. Therefore, predicting root position during orthodontic treatment is a critical factor for successful outcomes.

Two-dimensional (2D) images such as cephalometric and panoramic radiographs cannot evaluate the three-dimensional (3D) position of the teeth and roots relative to the maxillofacial region and alveolar bone during orthodontic treatments. With its increasing availability, cone-beam computed tomography (CBCT) has been used extensively in various fields of dentistry including endodontics, orthodontics, and oral surgery for diagnosis of complicated anatomy and treatment plans for complex rehabilitation [14,15]. For orthodontic treatment, the advantage of CBCT is that it provides the exact 3D location of the crown and roots of teeth and their relationship with both neighboring teeth and alveolar bone [16,17]. Therefore, visual and timely evaluation of root positioning using 3D imaging by CBCT is crucial in orthodontic treatments. However, CBCT is not without its disadvantages.

Since the effective radiation dose from CBCT is significantly higher compared to conventional radiographs, routine usage of CBCT is not recommended during orthodontic treatments as a substitute for conventional radiographs, especially when the population for orthodontic treatment is relatively young [18,19]. Furthermore, CBCT rendering of teeth lacks a precise occlusal surface and accurate interdigitation [20]. Artifacts from metal restorations and orthodontic brackets can also result in discrepancies [21]. On the other hand, digital scanning can provide precise tooth morphology and register accurate interocclusal relationship. Studies have also shown that brackets did not affect the accuracy of digital scans [22,23]. Therefore, registration and subsequent superimposition of CBCT and digital scans obtained with either an intraoral scanner or an extraoral lab scanner have become a standard workflow in many orthodontic appliance systems to assess tooth alignment. While this method is generally clinically acceptable, the registration of CBCT to digital scans is less accurate compared to registration of digital scans to digital scans [24].

To overcome these shortcomings, in 2014, Lee et al. introduced a monitoring method by creating “teeth composites” whereby the 3D digital model was composed of crowns extracted from digital scans and the root portion extracted from CBCT [24]. The authors demonstrated in an ex-vivo typodont study that this method was reliable to track the 3D position of whole teeth including roots. This method was not further verified in clinical studies due to impracticality related to the technique-sensitive, complex, and time-consuming process of threshold segmentation of teeth in real patients.

With the rapid development of imaging and digital technology in recent years, much improvement has been made in both hardware and software. Many commercial companies now provide service to clinicians to process CBCT and digital scan data and to produce 3D models with 3D printing. The cost of these services has also become significantly lower. Creating 3D digital crown/root models (3DCRM) by integrating digital scan crowns and CBCT root with patient data has become a feasible approach. Therefore, this method first introduced by Lee et al. in 2014 will be an ideal modality.

This is a retrospective study using clinical cases, and the aims of this study were (1) to assess the accuracy of this new method of predicting post-movement root position compared to the current method of using post-movement CBCT, (2) to analyze the association of accurately predicting the root position in each of the three tooth types, and (3) to analyze the association between the accuracy of the test method and the amount of actual root apex movement. We hypothesize that the 3DCRM method will be as accurate as the use of post-movement CBCT in evaluating root positioning and thus will eliminate the use of a second or multiple CBCTs during or at the completion of orthodontic treatment.

## 2. Materials and Methods

### 2.1. Data Collection

In five orthodontic cases, the pre- and post-movement CBCT images and scanned dental arches were obtained from the patient database of the Division of Orthodontics, Department of Developmental Oral Health Science, School of Dentistry, Iwate Medical University in Japan. The cases included received non-extraction orthodontic treatment. Cases with cleft-palate and other craniofacial syndromes as well as systemic diseases that could affect bone metabolism were excluded. This study was approved by the institutional review board (IRB) of Iwate Medical University, School of Dental Medicine (No. 01329).

The CBCTs were captured at 90–120 kV, 6.0–7.5 mA using a dental CBCT scanner (3D Accuitomo 170, J. MORITA CORP, Kyoto, Japan). The images were reconstructed with 0.28-mm-slice thickness and exported as digital imaging and communications in medicine (DICOM) files. Alginate impressions (Algiace Z, Sankin, Kogyo, Tokyo, Japan) of patients’ dental arches were poured in high-strength dental stone (New PlastoneII white, GC, Tokyo, Japan). The pre- and post-movement stone casts were scanned via an extraoral 3D digital scanner (MDS500 Dental Scanner, AGE Solutions S.r.l., Pisa, Italy) and exported as STL files.

### 2.2. Fabrication of 3D Digital Crown/Root Models (3DCRM)

**Figure 1:**
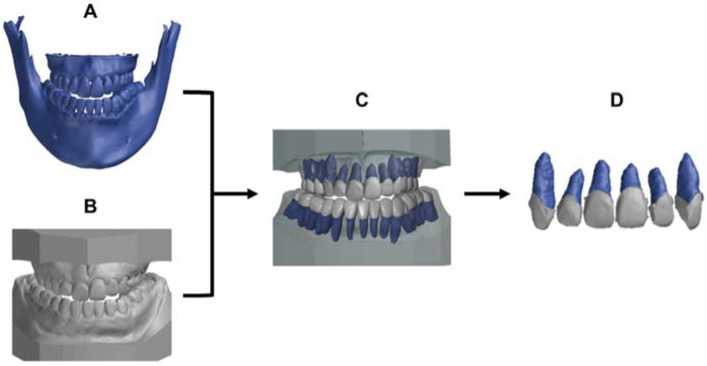
Protocol to generate the 3D digital crown/root models (3DCRM): (**A**), pre-movement CBCT image. (**B**), pre-movement scanned dental arches. (**C**), the pre-movement CBCT image was superimposed on the pre-movement scanned dental arches with the crown shape as an index. (**D**), the individual 3DCRMs of the six maxillary teeth were extracted from surrounding structures.

The CBCT DICOM files as well as digital scanning STL files were sent to a commercial 3D service company (3DDX, Boston, MA, USA) for further processing. Briefly, the pre- and post-movement DICOM files were imported into dental implant planning software (Simplant, Materialise Dental NV, Leuven, Belgium) and converted to stereolithography (STL) files. The 3D digital crown/root models (3DCRM) were created using pre-movement CBCT images and scanned dental arches (Figure 1). The STL data of the pre-movement CBCT images and the scanned dental arches were superimposed based on as many corresponding points as possible of the crowns (Figure 1A). Second, using the superimposed images, individual 3DCRMs of the six maxillary anterior teeth were created and exported as STL files (Figure 1B). The accuracy of the resulting 3DCRMs was checked by three clinicians before approval for use in the study.

### 2.3. Test Method to Predict the Post-Treatment Root Position

**Figure 2:**
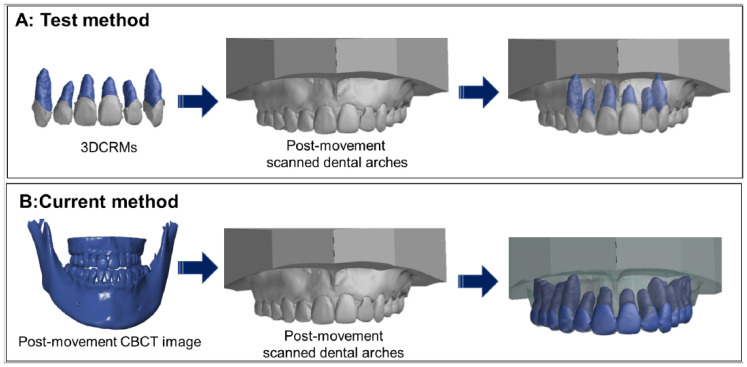
Determination of the post-movement root position by the test method and the current method. (**A**): The test method; the individual 3DCRMs were superimposed on the post-movement scanned dental arches with the crown shape as an index. (**B**): The current method; post-movement CBCT data were superimposed on the post-movement scanned dental arches.

These 3DCRMs were superimposed onto the post-movement scanned dental arches based on corresponding points of the crowns using 3D data inspection software (GOM inspect, GOM, Braunschweig, Germany, Figure 2A). The 3D coordinates on the X, Y, and Z axes of the root apex were measured by the same person three times at three different time points, and the average value was used for the analysis.

### 2.4. Current Method to Identify the Post-Treatment Root Position

The post-movement of CBCT and scanned dental arches were imported into 3D data inspection software, and these data were superimposed with the corresponding points of the crowns. The 3D coordinates on the X, Y, and Z axes of the root apex were measured in the same manner as that used for the test method (Figure 2B).

### 2.5. Analysis of Accuracy for Prediction of Post-Movement Root Position and Statistical Analysis

- Color displacement map

A color displacement map of the root was used by 3D data inspection software to quantify the differences between the root position created by the test method and the current method. The average displacement of each tooth was calculated, and the statistical differences among the tooth types were analyzed using one-way analysis of variance (ANOVA) (*p* < 0.01, SPSS ver. 24, IBM, Armonk, NY, USA).

- 3D coordinate assessment of the discrepancy in six directions.

The discrepancy between post-movement root position determined by the test method and that of the current method was calculated on the 3D axes X (DX), Y (DY), and Z (DZ) by the following Equation (1):

(1) $$\begin{array}{cc} \mathrm{DX} & =X_{test}-X_{current}\;(\mathrm{Positive}:\;\mathrm{to}\;\mathrm{labial},\;\mathrm{Negative}:\;\mathrm{to}\;\mathrm{palatal})\\ \mathrm{DY} & =Y_{test}-Y_{current}\;(\mathrm{Positive}:\;\mathrm{to}\;\mathrm{mesial},\;\mathrm{Negative}:\;\mathrm{to}\;\mathrm{distal})\\ \mathrm{DZ} & =Z_{test}-Z_{current}\;(\mathrm{Positive}:\;\mathrm{to}\;\mathrm{apical}-\mathrm{intrusion},\;\mathrm{Negative}:\;\mathrm{to}\;\mathrm{incisal}-\mathrm{extrusion}) \end{array}$$

The discrepancy on each of the three axes amongst the three tooth types was compared using one-way ANOVA and Tukey’s post-hoc test (*p* < 0.01). A one-sample *t*-test was also performed to compare the discrepancy on each of the three axes and the CBCT voxel size.

- Association between the accuracy of the test method and the amount of actual root apex movement.

The amount of actual root apex movement (DARAM) was calculated using the 3D coordinates of pre-movement of CBCT (Pre) and post-movement of CBCT (Post) using Equation (2):

(2) $$\mathrm{DARAM}\;\left(X\right)=\left|X_{Post}-X_{Pre}\right|\;\mathrm{DARAM}\;\left(Y\right)=\left|Y_{Post}-Y_{Pre}\right|\;\mathrm{DARAM}\;\left(Z\right)=\left|Z_{Post}-Z_{Pre}\right|$$

Pearson’s correlation coefficient between the distance of root movement (DARAM on the X, Y, and Z axes) and the absolute value of the discrepancy on the X, Y, and Z axes was calculated.

## 3. Results

### 3.1. Displacement on the Color Map

**Figure 3:**
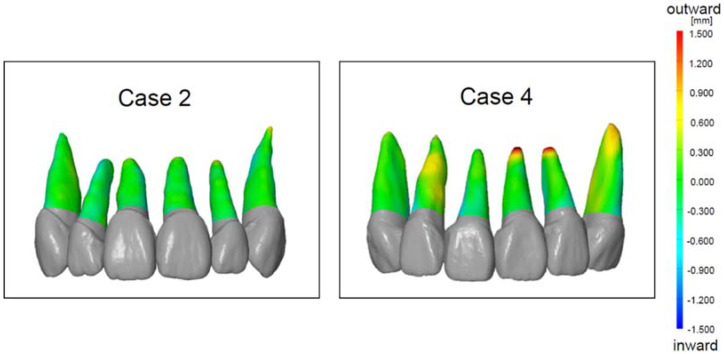
The color displacement map of the root position of case 2 and case 4. The green (zero point) indicates that the test method and the current method had no displacement. The red indicates the outward displacement of the test method compared to the current method. The blue indicates the inward displacement of the test method compared to the current method.

The color displacement map of the root position based on the test method and the current method displayed three colors: green, blue, and red (Figure 3). Case 2 was predominantly in the green range, indicating a minimum discrepancy between the test method and the current method. In contrast, case 4 had a significant yellow–red color at the root apex, indicating outward displacement greater than 0.5 mm (Figure 3). The average root displacement of five cases was −0.16 ± 0.05 mm, and there was no significant difference among the three tooth types (*p* > 0.01, Table 1).

### 3.2. Discrepancy Based on 3D Coordinates

**Figure 4:**
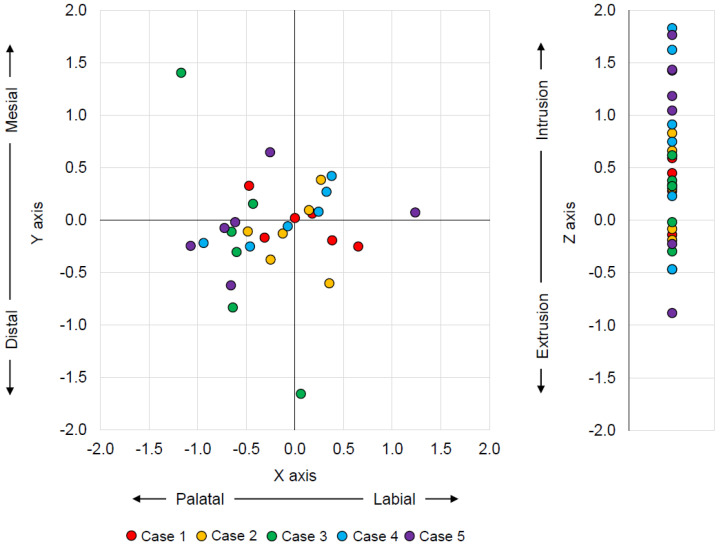
The distribution of the discrepancy between the test method and the current method on the X, Y, and Z axes with zero point as the current method. The X axis represents labial–palatal, the Y axis mesial–distal, and the Z axis apex/intrusion– incisal/extrusion discrepancies.

**Figure 5:**
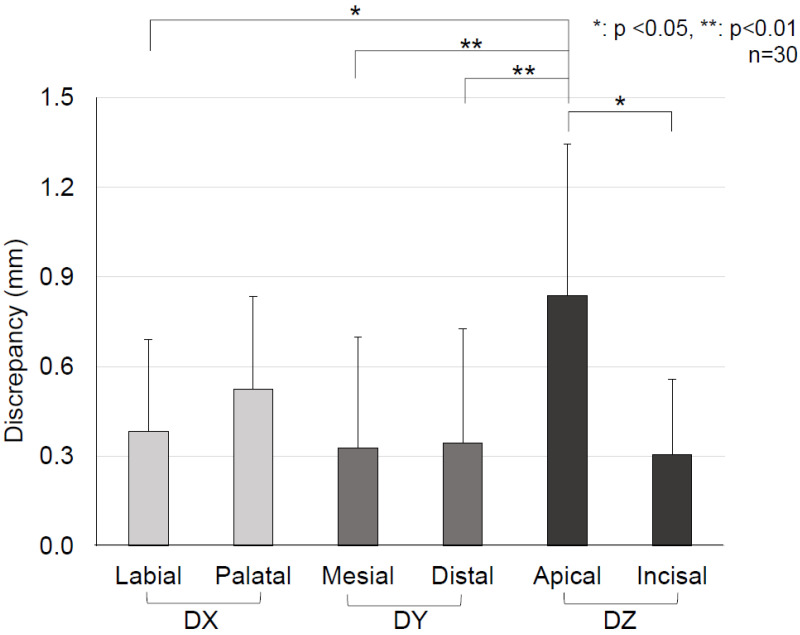
Comparison of the average discrepancy of each X, Y, and Z axis and six directions based on one–way ANOVA and Turkey’s post-hoc test. The discrepancy on the Z axis in the apical direction (intrusion direction) was significantly greater than that observed for all other directions (*p* < 0.05).

Figure 4 shows the distribution of discrepancy on the X (the labial and palatal directions), Y (the mesial and distal directions), and Z (the intrusion and extrusion directions) axes with the zero point, which is the location determined by the current method. On the XY coordinate plane, mesial–palatal discrepancy is shown in quadrant I, mesial–labial discrepancy in quadrant II, distal–labial discrepancy in quadrant III, and distal–palatal discrepancy in quadrant IV. The greatest discrepancy was seen in quadrant III, which means that the root position was predicted to be more distal and labial. The Z axis analysis indicated that root position was overly predicted in the apical direction. The average discrepancy of each of the X, Y, and Z axes and directions is shown in Figure 5. The discrepancy on the Z axis in the apical direction (intrusion direction) was significantly greater than that for all other directions for all tooth types (*p* < 0.05, Figure 5). The discrepancy on the Z axis in the apical direction was also significantly higher than the CBCT voxel size (*p* < 0.01), but no significant difference was observed between the discrepancy on the other five axes and the CBCT voxel size. The average discrepancy of each of three tooth types is shown in Table 2. No significant difference was observed among the tooth types on the X, Y, and Z axes.

### 3.3. Association

**Figure 6:**
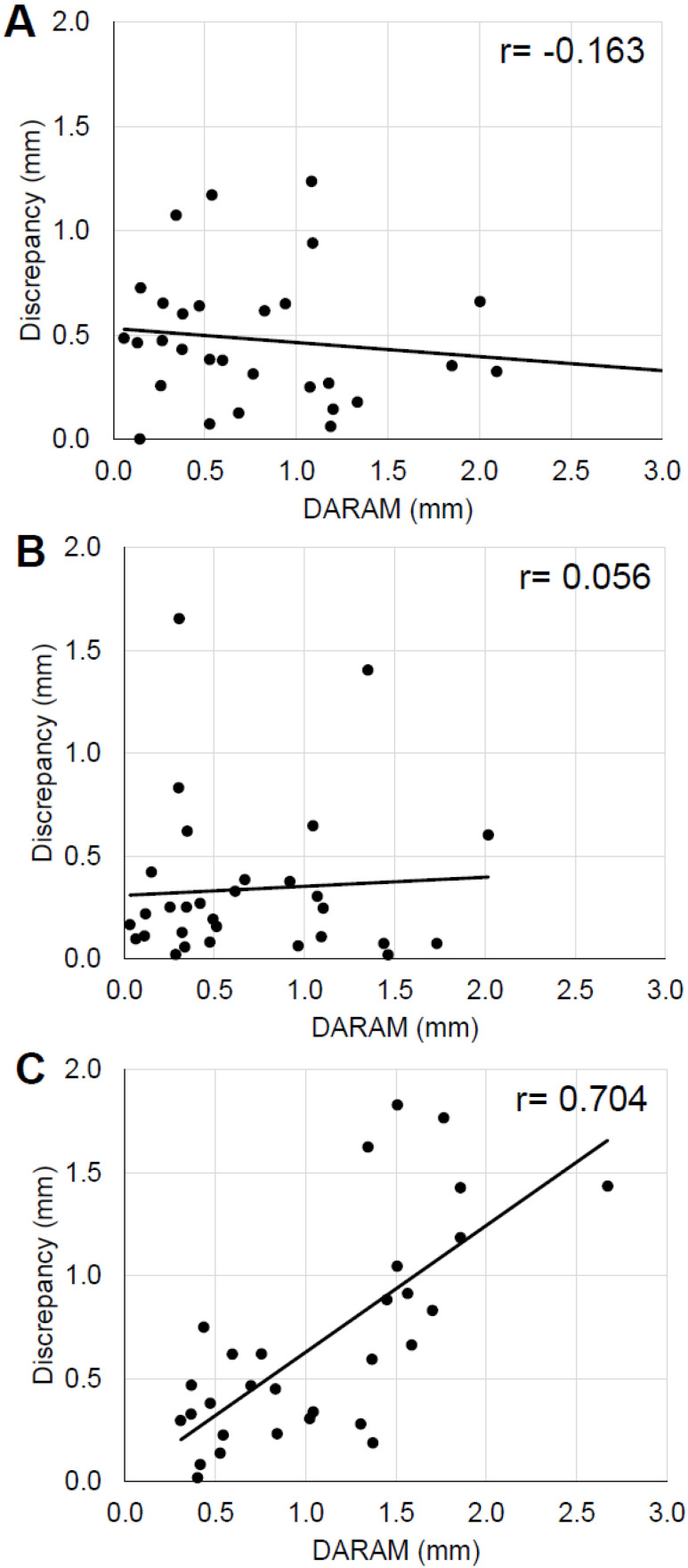
Correlation between the actual amount of root apex movement (DARAM) and the discrepancy absolute value. (**A**), X axis (**B**), Y axis, (**C**), Z axis.

There was a strong positive correlation (*r* = 0.71) between the amount of root movement on the Z axis (DARAM) and the absolute value of discrepancy on the Z axis. This means that the prediction accuracy of root position by the test method decreased as the distance of root movement increased on the Z axis. No correlation was observed for the X and Y axes (Figure 6).

## 4. Discussion

Orthodontic treatment aims to move teeth to ideal positions within the extent of the alveolar bone without damaging the roots or adjacent structures [17]. Therefore, it is important to monitor the position of both the roots and crowns of teeth. The CBCT and panoramic radiographs are used to confirm the root position during orthodontic treatments. The majority of orthodontists use panoramic radiographs to visualize the correct root positions. A survey in 2008 found that 67.4% and 80.1% of American orthodontists took panoramic radiographs during orthodontic treatments and post-treatment, respectively [25]. There is a report that the magnification–distortion ratio of the CBCT images (1.04) is smaller than that of panoramic radiographs (1.20) [26]. However, several papers reported that CBCT should not be used routinely on every patient due to resulting higher doses of radiation in comparison to conventional radiographs [18,19,27,28]. Therefore, our new method to accurately predict the root position during orthodontic treatments without taking additional CBCT images will be beneficial and necessary.

The 3DCRM used in this study is a digital model of the crown and root created by the pre-movement scanned dental arches and CBCT images. We hypothesized that the root position during orthodontic treatments could be predicted by superimposing 3DCRM onto the post-movement scanned dental arches without extra CBCT images. The 3DCRM can be individually superimposed on each tooth of post-movement scanned dental arches by utilizing the crown portion as the index.

In this study, we evaluated the accuracy of the new method (test method) in predicting root position using color mapping analysis and 3D coordinate analysis. The advantage of the color mapping analysis is to visualize the displacement of the entire root surface.

The color mapping analysis visually indicated the distinguishable displacement between the test method and current method mainly at the root apex and the rest root surface had minimal discrepancy within the green level. Furthermore, our results (average 0.16 ± 0.05 mm displacement) based on clinical cases are comparable to results from the ex vivo typodont study by Lee et al. in which displacements of 0.17 ± 0.32 mm and 0.11 ± 0.16 mm were found for the maxillary and mandibular teeth, respectively [24].

3D coordinate analysis can provide an accurate prediction of the root apex in the direction of discrepancy with numerical data. The 3D coordinate analysis indicated a significant discrepancy only on the Z axis (the apex/intrusion and incisal/extrusion directions), i.e., not on the X axis (the labial and palatal directions) or the Y axis (the mesial and distal directions). The numerical data of the average discrepancy on the Z axis–apical were 0.83 mm, which is 2.9 times greater than a single CBCT voxel unit (0.28 mm). In contrast, the data on the X and Y axes were about 1.1–1.8 times greater without statistical difference. This means that a discrepancy existed with regards to the root apex position on the Z axis, especially in the intrusion direction. In addition, a strong positive correlation was observed between the distance of the actual root apex movement and the discrepancy along the Z axis.

The discrepancy in the apical direction is likely due to root resorption that occurs during orthodontic treatment. Based on the changes in the crown orientation, superimposition of the pre and post-movement scans allowed the root orientation, and thus the bucco–lingual and mesio–distal orientations could be predicted. However, resorption of the apex of the root and the amount of resorption cannot be predicted, leading to the discrepancy along the Z axis. Excessive orthodontic and occlusal-loading forces are associated factors of root resorption [29,30,31,32,33,34,35]. Since the root resorption associated with orthodontic treatment can occur and progress without any symptoms, monitoring root positions with radiographs during orthodontic treatment is essential [36]. Routine radiographic evaluation using periapical films is recommended to accurately monitor the root position during orthodontic treatment.

The limitations of this study are the small sample sizes and the lack of variation of clinical cases. The cases used were non-extraction cases, and the amount of root movement was relatively small. Further study is required involving larger samples sizes and moderate to severe crowding cases to validate this method.

## 5. Conclusions

The 3DCRM method showed promising potential to accurately predict root position during orthodontic treatments. However, in cases of root resorption, it was difficult to identify the position of the root in in the apico–coronal direction. Therefore, frequent assessment of root resorption using periapical radiographs is recommended to compensate for the lack of accuracy of predicting movement along the Z axis.

## Figures and Tables

**Table 1:** The average root displacement of each tooth type.

	Average	S.D.	Max.	Min.
Total	−0.165	±0.053	1.889	−1.222
Central incisor	−0.140	±0.102	1.889	−1.222
Lateral incisor	−0.136	±0.122	1.878	−1.000
Canine	−0.177	±0.124	1.516	−1.055

Unit: mm.

**Table 2:** The average root displacement of each tooth type on the X, Y, and Z axes and in six directions. There was no significant difference among the three tooth types.

Discrepancy		Central Incisor		Lateral Incisor		Canine	
Axis	Direction	Mean (SD)	*n*	Mean (SD)	*n*	Mean (SD)	*n*
X axis	Palatal	0.63 (0.38)	6	0.46 (0.28)	10	0.51 (0.18)	3
	Labial	0.48 (0.44)	4	NA	0	0.33 (0.16)	7
Y axis	Distal	0.18 (0.06)	5	0.24 (0.27)	7	0.61 (0.50)	6
	Mesial	0.41 (0.51)	5	0.17 (0.13)	3	0.35 (0.21)	4
Z axis	Incisal	0.27 (0.19)	2	0.21 (0.02)	2	0.36 (0.30)	5
	Apical	0.92 (0.61)	8	0.83 (0.47)	8	0.72 (0.32)	5

Unit: mm.

## Data Availability

Not applicable.
